# Antimicrobial Peptides as Part of the Arsenal of Constitutive and Inducible Seed Defences in Tomato Seed Exudates Against Pathogens

**DOI:** 10.1111/mpp.70164

**Published:** 2025-10-27

**Authors:** Łukasz P. Tarkowski, Benjamin Hubert, Muriel Marchi, Camille Tranchant, Stéphanie Boutet, Céline Brosse, Mathilde Causse, Thierry Balliau, Mélisande Blein‐Nicolas, Massimiliano Corso, Sébastien Aubourg, Jérôme Verdier

**Affiliations:** ^1^ University of Angers, Institut Agro, INRAE, IRHS, SFR QUASAV Angers France; ^2^ Université Paris‐Saclay, INRAE, AgroParisTech, Institute Jean‐Pierre Bourgin for Plant Sciences (IJPB) Versailles France; ^3^ Génétique et Amélioration Des Fruits et Légumes, Centre de Recherche PACA, INRAE, UR1052, CS60094 Avignon France; ^4^ GQE—Le Moulon Université Paris‐Saclay, INRAE, CNRS, AgroParisTech Gif‐sur‐Yvette France

**Keywords:** seed exudates, seed immunity, seed pathogens, tomato

## Abstract

Seed immune responses are an underexplored area in host–pathogen interactions, leaving seed–pathogen interactions poorly understood despite their considerable economic impact. This study examined tomato seed defences by assessing the antimicrobial activity (AA) of seed exudates during germination. Results showed genotype‐dependent and constitutive defence responses from seeds showing AA in exudates. Seed priming with a panel of elicitors such as methyl jasmonate (MeJA) enhanced AA in certain genotypes, highlighting an inducible defence response. Both constitutive and inducible (elicitor‐dependent) seed defences were genotype‐dependent and more effective against the non‐host pathogen *Alternaria brassicicola* (Abra43), while host pathogens seemed resistant to exudates' AA, suggesting that they developed strategies to neutralise exudates' AA. Multi‐omic analyses revealed distinct hormonal and molecular pathways involved in constitutive and inducible defences. By characterising exudates and correlating genotype‐ and elicitor‐specific AA, candidate antimicrobial compounds were identified. As proof of concept, we functionally validated the AA of a putative defensin (Solyc07g007755) whose expression was highly correlated with the observed AA of the seed exudate against Abra43, demonstrating the potential of our dataset for the development of phytosanitary strategies to protect seeds during germination.

## Graphical abstract

Tomato seeds possess genotype‐dependent constitutive and inducible antimicrobial defences, enhanced by elicitors like methyl‐jasmonate, including antimicrobial molecules, offering insights for improving seed protection strategies.

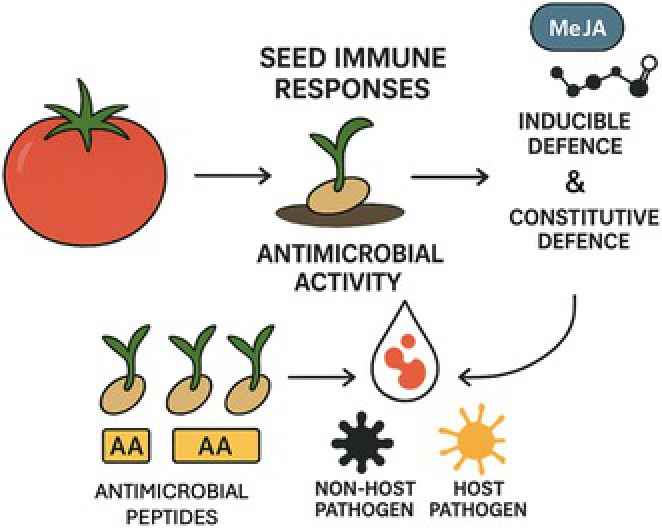

## 1 Introduction

Global food security is currently facing unprecedented challenges arising from climate change, human population growth and geopolitical instability, making the need to decrease the yield gap an imperative. Pathogen attacks account for a considerable part of global yield losses, with current models predicting the situation to get worse due to climate change‐related factors (Ficke et al. 2018; Savary et al. 2019; Singh et al. 2023). Although important progress in plant–pathogen interactions has been made in the last decades, current crop disease management still relies heavily on the use of chemical pesticides (Sharma et al. 2019). Therefore, the development of sustainable alternatives is an unavoidable requirement for current crop research programmes. Several research leads have been proposed in this regard, such as exogenous application of natural elicitors, inoculation with beneficial microbes, or manipulation of the soil microbiome (Syed Ab Rahman et al. 2018; Pascale et al. 2020; Desmedt et al. 2021). Such strategies were mostly developed for and applied on adult plants, targeting vegetative tissues. In contrast, research on the interactions between pathogens and reproductive tissues such as seeds has received scarce attention to date, even though it can be argued that seeds are one of the most valuable components of plants' fitness and the seed stage represents one of the most susceptible segments of the plants' lifecycle (Dalling et al. 2011). On the one hand, seeds are a fundamental vehicle for pathogen dispersal and multiplication, making successful pathogen transmission from mother plants (vertical transmission) or from the surrounding environment (horizontal transmission) a major determinant of pathogens' fitness and determining future disease outbreaks on adult plants (Darsonval et al. 2009; Darrasse et al. 2010; Denancé and Grimault 2022). On the other hand, seed pathogens can directly affect seed health, causing seed rot and/or seed germination failure, known as damping‐off, directly impacting seedling establishment rates and leading to up to 80% of field yield loss depending on environment, plant genotype and soil (Lamichhane et al. 2017; Dell'Olmo et al. 2023). Taking these factors together, it can be concluded that (a) there is a necessity to fill the current knowledge gap about seed immune responses and seed–pathogen interactions; and (b) sustainable pest management strategies should consider the seed stage as a research target. For the first point, the primary limitation for the study of seed–pathogen interactions is likely the difficulty to set up a pathosystem with seeds, as infection symptoms are rarely visible and hard to quantify and destructive methods are required to analyse endophytic pathogens (Faeth and Fagan 2002; Dutta et al. 2014; Ortega‐Cuadros et al. 2022; Dell'Olmo et al. 2023). Concerning the second point, seed coating with pesticides remains a common practice to control seed sanitary quality (Hitaj et al. 2020). This technique is being increasingly criticised due to the high cost of the equipment required to coat seeds, its negative off‐target effects against other microorganisms and the chemical nature of several coating compounds (Pedrini et al. 2017; Gross et al. 2020; Hitaj et al. 2020). Other common phytosanitary strategies to control microbial pathogens include seed disinfection through heat treatment with steam or hot water, or cultural practices such as crop rotation, aimed at decreasing soil disease conductivity (Keler et al. 2020; Kim et al. 2022).

There are very few examples of sustainable pest control strategies being applied on seeds. Seed priming designs a group of methodologies aimed to ameliorate seed physiological parameters such as germination rate and/or improve abiotic stress tolerance by soaking seeds in specific solutions before sowing, with typical seed priming techniques including hydropriming (soaking in water) and osmopriming (soaking in high osmolarity solutions) (Paparella et al. 2015). Several attempts to prime seeds with natural elicitors (known as seed biopriming or seed defence priming) have been described and correspond to the classic defence priming approach (Martinez‐Medina et al. 2016; Talavera‐Mateo et al. 2023), with several other studies that are likely not published due to private intellectual property (Pedrini et al. 2017). However, the evaluation of resistance induction has been done on germinated seedlings or adult plants due to the impossibility to set up a seed‐based pathosystem (Worrall et al. 2012; Song et al. 2017). If such studies show that seeds are indeed receptive to elicitors, they open the question about the molecular nature of seed defence pathways. Indeed, detailed molecular characterisation of the events triggered by seed defence priming (e.g., through‐omics techniques) is currently lacking.

**FIGURE 1:**
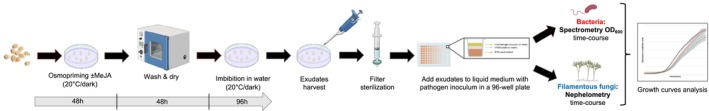
Representation of the workflow used for seed priming, seed exudates production and microbial growth assays. The grey arrow illustrates the timing of the seed priming and exudate production. See the Section Experimental Procedures for details.

Here, we characterised seed defence pathways at germination by hypothesising the existence of an inducible component besides a constitutive one. To study the constitutive component, we selected three tomato ( *Solanum lycopersicum* ) genotypes (Stupicke, Criollo and Micro‐Tom). In regard to the inducible component, we used the seed defence priming approach by adding elicitors to osmopriming solutions (Figure 1). Although hormonal elicitors will have obvious negative effects on the growth (germination)–defence trade‐off, our main goal was rather to characterise the hormonal‐elicited pathways and identify novel molecules produced following such treatments that may have protective roles against seed pathogens. To characterise the defensive role of seed‐produced compounds, we took advantage of a recently developed protocol to study the antimicrobial activity (AA) of tomato seed exudates towards a panel of seed pathogens (Hubert, Leprince, et al. 2024). These data were complemented by transcriptomic and untargeted metabolomic analyses on seeds and/or seed exudates to identify candidate molecular mechanisms underlying the observed AA. Results obtained revealed the presence of genotype‐dependent constitutive defences as well as elicitor‐induced defences in exudates. We observed that both genotype‐ and elicitor‐dependent effects were effective against the incompatible fungal pathogen *Alternaria brassicicola*. The panel of detected metabolites and genes associated with exudates AA can be used as a foundation for the development of sustainable strategies focused on the seed stage, besides providing novel leads to further investigate pathogen defence mechanisms in seeds. We exemplified this concept by showing that a putative defensin encoded by one of the genes correlated with exudates' AA displayed a strong AA against *A. brassicicola*.

## 2 Results

### 2.1 Analysis of the Antimicrobial Effect of Tomato Seed Exudates Against a Panel of Pathogens Reveals Genotype‐Specific Activities

**FIGURE 2:**
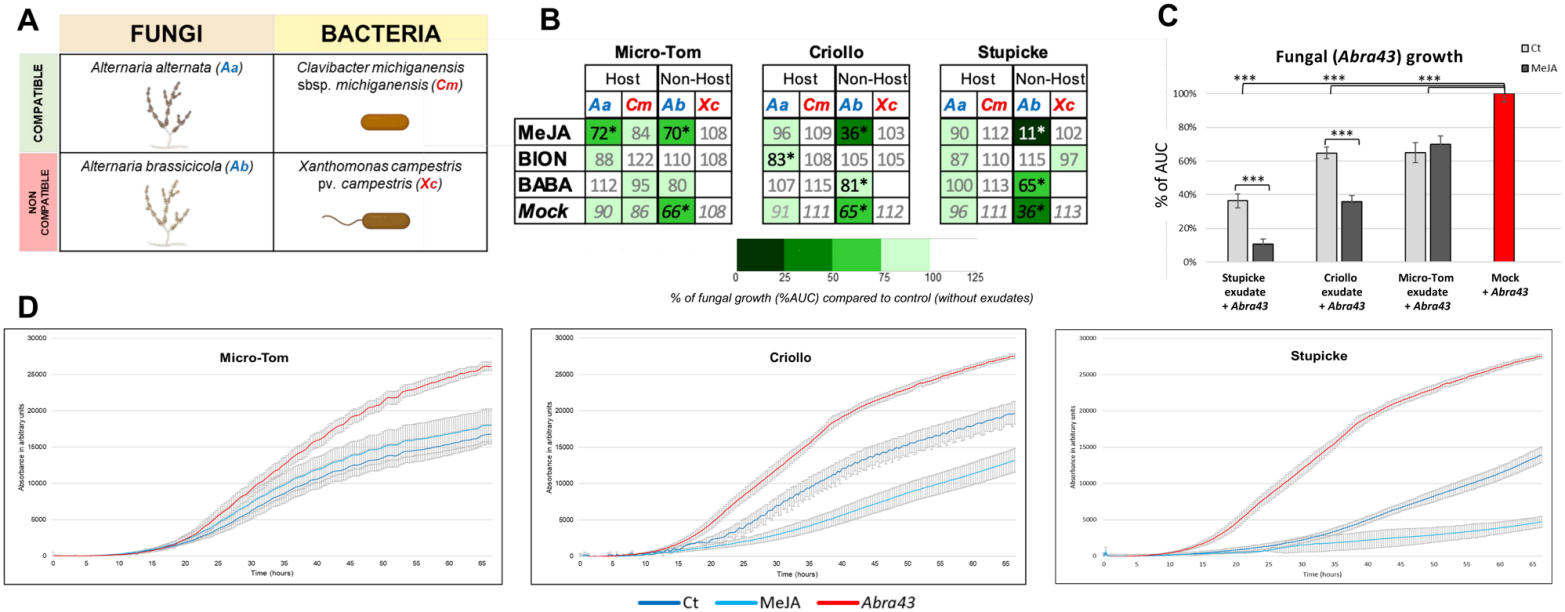
Effect of exudates from osmoprimed tomato seeds on the growth of plant pathogens in liquid culture. (A) Matrix representing the plant pathogens selected for this study. (B) Heatmap showing the microbial growth in presence of the seed exudates from different genotypes and the priming agents. Numbers represent the percentage of the total area under the curve (AUC) compared to the control without exudates, which is used as a proxy for fungal growth. Aa, *Alternaria alternata* ; Ab, *Alternaria brassicicola*; Cm, *Clavibacter michiganensis* subsp. *michiganensis*; Xc, *Xanthomonas campestris* pv. *campestris*. Blue letters indicate fungi and red letters indicate bacteria. Asterisks indicate that the condition resulted in a significant decrease of microbial growth compared to control without exudates. (C) Histograms representing growth inhibition of *A. brassicicola* (strain Abra43) in presence of exudates from methyl jasmonate (MeJA)‐primed seeds. Values are expressed as percentages of the total AUC compared to the untreated control. Ct, mock‐primed seeds; MeJA, MeJA 2 mM‐primed seeds. Asterisks indicate significant differences according to Student's *t* test (***p < 0.001). Error bars represent SD. (D) Curves showing the growth of *A. brassicicola* (strain Abra43) in presence of exudates from MeJA‐primed seeds. *y*‐axis: Absorbance values in arbitrary units; *x*‐axis: Time in hours. Data were analysed and plotted with the Mars Omega software (v. 3.42, BMG LabTech). Error bars represent SD.

Based on preliminary results (Bizouerne et al. 2023), we selected two tomato cultivars for our study from the Multi‐allelic Genetic InterCross (MAGIC) population (Pascual et al. 2015): a small‐fruit cultivar (Criollo) and a large‐fruit cultivar (Stupicke). We added to this panel the reference genotype Micro‐Tom (Shikata and Ezura 2016). Data about pathogen susceptibility are not available for Stupicke and Criollo, while Micro‐Tom is generally regarded as a susceptible cultivar (Takahashi et al. 2005). First, we explored the AA in seed exudates of the three cultivars against a panel of four seed pathogens with respect to two factors: their biology (fungal vs. bacterial) and their ability to infect the selected host plant (compatible vs. incompatible interactions). A compatible pathogen can infect and trigger disease in the host while an incompatible pathogen cannot (Glazebrook 2005) (Figure 2A). Two fungi and two bacteria were selected. The fungi *Alternaria alternata* and *Alternaria brassicicola* (henceforth referred to as its strain name Abra43, Pochon et al. 2012), respectively involved in a compatible and incompatible interaction with tomato, were used. The bacteria *Clavibacter michiganensis* and *Xanthomonas campestris* , similarly inducing compatible and incompatible responses in tomato, were selected as relevant seed pathogens (Pochon et al. 2012; Meena et al. 2013; Tancos et al. 2013; Chen, Ruh, et al. 2021; Chen, Zhang, et al. 2021) (Figure 2A).

Exudates from tomato seeds primed with an osmotic solution without any elicitors (mock) were first tested to evaluate the constitutive AA in seed exudates of different genotypes. Profiling fungal growth (nephelometry) and bacterial growth (spectrometry) (Figure 1) showed that the exudates of non‐primed seeds from the three tomato genotypes exhibited no detectable antibacterial effect (Figure 2B). In contrast, strong antifungal activities were detected in the exudates of Micro‐Tom, Criollo and Stupicke in the presence of the non‐host pathogen, Abra43, with a fungal growth reduction of 45%, 64% and 45% compared to mock, respectively. These results indicate that primed seeds were able to externally secrete molecules with antifungal or fungistatic activities (Figure 2B), displaying a genotype‐dependent degree of AA.

### 2.2 Analysis of the Antimicrobial Effect of Tomato Seed Exudates Against a Panel of Pathogens Reveals Both Genotype‐ and Elicitor‐Specific Activities

To investigate whether seeds AA have an inducible component, we measured the AAs of seed exudates against our panel of pathogens after defence elicitation applied during a 48 h osmopriming treatment (Figure 1). The tested elicitors were methyl jasmonate (MeJA at 2 mM final concentration) to stimulate the jasmonic acid (JA) pathway, Bion (composed of 50% acibenzolar‐*S*‐methyl, a commercialised analogue of the phytohormone salicylic acid [SA], at 10 mM) to stimulate the SA pathway, and β‐aminobutyric acid (BABA at 10 mM) as a third relevant defence pathway in the context of defence priming (Luna et al. 2014; Yan and Dong 2014; Wan and Xin 2022).

We tested the AAs of the exudates of the three genotypes with seed elicitation against our four seed pathogens panel. Statistical analyses showed a weak effect of Bion elicitation on the AA of elicited seeds against *A. alternata* growth (with a growth reduction of about 17%), exclusively in Criollo seed exudates (Figure 2B). Similarly, BABA‐treated seeds produced exudates showing a reduction of 19% and 35% of Abra43 growth from Criollo and Stupicke seeds, which were surprisingly lower than AAs of mock‐treated seeds in both genotypes. These results suggested the activation of antagonist defence pathways in mock‐ and BABA‐treated seeds. In the case of MeJA‐treated seeds, we observed an additional increase in AA compared with mock‐treated seeds, at the expense of seed germination vigour (Figure S1). Concerning Abra43, we already observed an AA following mock treatment in the seed exudates of the three genotypes, which was enhanced in a statistically significant manner following MeJA treatment in Criollo and Stupicke to reach 64% and 89% of fungal growth reductions (Figure 2B), starting from 35% and 64% in mock‐treated seeds, respectively. In contrast, no additional effect of MeJA treatment on the AA of Micro‐Tom exudates against Abra43 was observed compared to mock‐treated Micro‐Tom seeds (Figure 2B). We also observed a reduction of *A. alternata* growth by 28% in MeJA‐elicited Micro‐Tom exudates.

These contrasting results of AAs of seed exudates between genotypes, between plant resistance inducers (PRIs) and against different pathogens illustrate the complexity of the interaction between PRIs, seeds and defence pathways, as described in other plant tissues and pathosystems (Thaler et al. 2012; Ullah et al. 2022). Based on these results, we further investigated the impact of constitutive and MeJA‐induced defence responses on Abra43. Although *A. brassicicola* is not a tomato pathogen, we anticipated that analysing such AA could preferentially lead to the potential discovery of protective molecules against non‐host pathogens.

### 2.3 Transcriptomic Analysis to Identify Constitutive and Inducible Seed Defences

**FIGURE 3:**
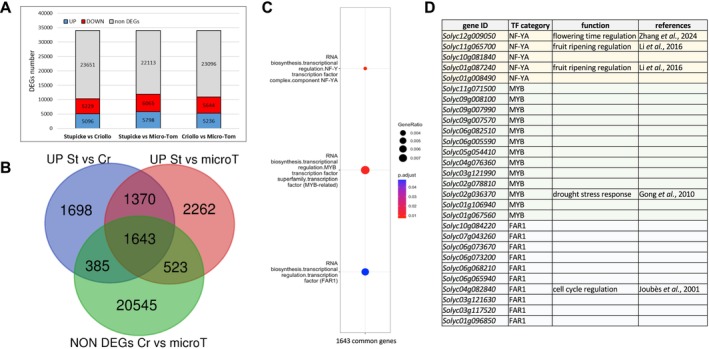
Transcriptomic analysis of the genotype effect on seeds. (A) Histograms summarising the total number of differentially expressed genes (DEGs) identified in the three pairwise comparisons. DOWN, downregulated DEGs; UP, upregulated DEGs. (B) Venn diagram built by using upregulated DEGs of the Stupicke versus Criollo comparison (UP St vs. Cr), the Stupicke versus Micro‐Tom comparison (UP St vs. microT), and the non‐DEGs of the Criollo versus Micro‐Tom comparison (Non‐DEGs Cr vs. microT). (C) Dot plots showing functional categories enriched in the 1643 common genes between the three subsets of the Venn diagram in panel (B). Significantly enriched categories were determined with the Benjamini–Hochberg test. Graphics were generated with the Clusterprofiler R package (v. 4.0). (D) Table showing the genes detected in the three clusters in panel (C), together with available information on their function and corresponding references. TF, transcription factor.

**FIGURE 4:**
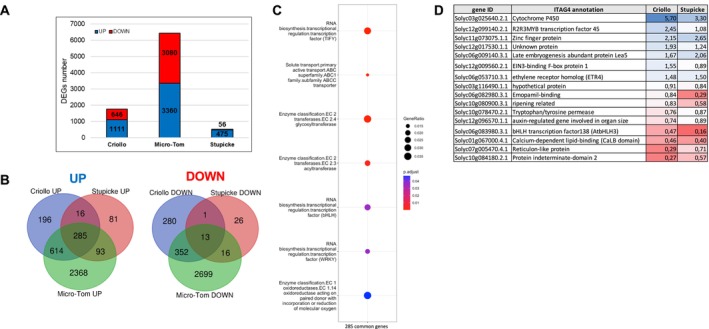
Transcriptomic analysis of the methyl jasmonate (MeJA) treatment on primed seeds. (A) Histogram summarising the number of detected differentially expressed genes (DEGs) in the RNA‐seq analysis by comparing MeJA‐primed versus mock‐primed seeds for the three genotypes. (B) Venn diagrams between the three genotypes showing the number of common and unique MeJA‐upregulated DEGs (UP, left diagram) and MeJA‐downregulated DEGs (DOWN, right diagram). (C) Dot plots showing functional categories enriched in the 285 commonly upregulated DEGs following MeJA priming in seeds of Criollo, Stupicke and Micro‐Tom cultivars. Significantly enriched categories were determined with the Benjamini–Hochberg test. Graphics were generated with the ClusterProfiler R package (v. 4.0). (D) Table showing the annotations of the 16 common MeJA‐upregulated genes between Stupicke and Criollo genotypes. ‘Criollo’ and ‘Stupicke’ columns contain log_2_fold change values for the two genotypes with their functional annotations from ITAG4.

To identify the molecular mechanisms contributing to constitutive defence against Abra43, we took advantage of the contrasting AA of seed exudates present in Micro‐Tom (34%), Criollo (35%) and Stupicke (64%) (Figure 2C,D). Pairwise comparisons of mock‐treated seed transcriptomes generated through RNA‐seq of the three genotypes were performed by placing the genotype with the higher AA first, and an adjusted *p*‐value threshold of 5% was set to identify differentially expressed genes (DEGs). We identified 10,325 DEGs (including 5096 upregulated genes in Stupicke) from Stupicke versus Criollo, 11,863 DEGs (including 5798 upregulated genes in Stupicke) from Stupicke versus Micro‐Tom and 10,880 DEGs from Criollo versus Micro‐Tom, with 23,096 non‐DEGs between these two genotypes, which displayed almost equal AA activity (Figure 3A, Table S1). To identify genes potentially involved in the constitutive defence process, a Venn diagram was generated with upregulated genes in Stupicke versus Criollo (as Stupicke showed a higher AA than Criollo), upregulated genes in Stupicke versus Micro‐Tom (as Stupicke showed a higher antimicrobial activity than Micro‐Tom) and the non‐DEGs between Micro‐Tom and Criollo (as Criollo showed similar AA to Micro‐Tom) (Figure 3B). We reasoned that, as there is no significant difference in AA between Criollo and Micro‐Tom, genes involved in the constitutive AA displayed by these two genotypes compared to mock should be present in the non‐DEGs subgroup. We revealed a set of 1643 DEGs (Table S1). An over‐representation analysis of this gene set highlighted three statistically enriched functional classes: NF‐YA transcription factors (TFs), MYB TFs and FAR1/FHY3 TFs (Figure 3C). Scarce information is available for the genes detected in these three clusters, with one NF‐YA TF being involved in flowering, two NF‐YA TFs in fruit ripening, one MYB TF involved in drought stress responses and one FAR1 TF involved in cell cycle regulation (Joubès et al. 2001; Gong et al. 2010; Li et al. 2016; Zhang et al. 2024) (Figure 4C).

Second, to elucidate the seed defence mechanism that could be elicited in seeds following the MeJA treatment, referred to as inducible seed defence, we analysed transcript expressions in our three seed genotypes after mock or MeJA treatment by performing pairwise comparisons. Different genotypes displayed different degrees of transcriptional changes following MeJA treatment with 1757, 531 and 6440 DEGs for Criollo, Stupicke and Micro‐Tom, respectively (Figure 4A, Table S2). A focus on upregulated genes following MeJA treatments revealed 285 genes commonly upregulated in all three genotypes (Figure 4B). *SlCEVI57*, encoding a proteinase inhibitor (Francia et al. 2007), is strongly induced by MeJA, displaying the highest log_2_ fold change values in both Stupicke (11.48) and Criollo (9.19). Moreover, five other genes encoding proteases and protease inhibitors are present in this list (Table S2). An over‐representation analysis of the commonly upregulated genes showed seven functional classes over‐represented in MeJA‐treated seeds (Figure 4C). Out of these seven functional classes, some are well‐known to contribute to plant immunity via JA signalling, such as WRKY TFs, bHLH TFs and TIFY TFs (Sasaki‐Sekimoto et al. 2013; Goossens et al. 2017). In particular, genes present in the upregulated gene ontology categories (Figure 4C) are known to be involved in immune processes in tomato, such as *SlWRKY37* and *SlWRKY39* (Sun et al. 2015; Wang et al. 2022) (Table S3). The 16 genes resulting from the Stupicke/Criollo intersection are of particular interest (Figure 4D), considering the gap of AA between these two genotypes and Micro‐Tom (Figure 1C). Genes associated with defence processes are present in this subgroup, such as ethylene signalling and perception (*Solyc06g053710*, *Solyc12g009560*). Other genes, such as *Solyc06g083980* and *Solyc12g099140*, encode TFs belonging to families known to be involved in defence activation (bHLH and R2R3MYB, respectively), representing potential candidates involved in the build‐up of the observed AA.

**FIGURE 5:**
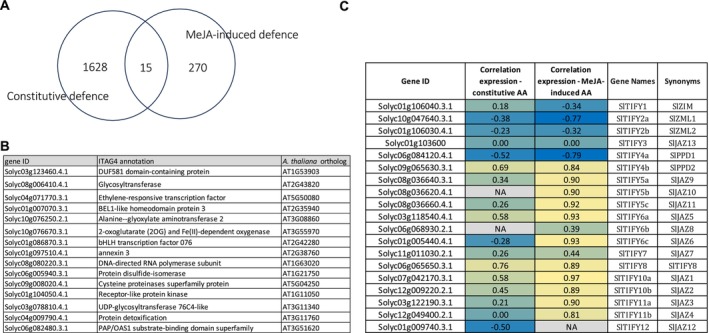
Common genes between constitutive and methyl jasmonate (MeJA)‐inducible defence. (A) Venn diagram showing the superposition between the subset of 1643 genes (Figure 3B) related to constitutive defence (‘Constitutive defence’) and the subset of 285 MeJA‐upregulated genes common between the three genotypes (Figure 4B) (‘MeJA‐induced defence’). (B) Table showing annotation details of the 15 common genes between Constitutive and MeJA‐induced defence subsets. The last column shows the name of the best homologue match in the genome of *Arabidopsis thaliana* according to ITAG4. (C) Table showing the Pearson coefficient correlation values between the JAZ/TIFY gene expressions and constitutive/MeJA‐induced antimicrobial activity (AA).

These data suggested that different hormonal defence pathways are involved in constitutive and inducible defence mechanisms. We further compared gene sets potentially involved in constitutive defence (1643 genes) and those involved in MeJA‐inducible defence (285 genes), showing a low overlap with only 15 in common, reflecting that constitutive and inducible defence mechanisms may function using different molecular pathways (Figure 5A,B). To further confirm this, we analysed the correlation of gene expressions of the 19 JAZs transcripts identified in the tomato genome (Chini et al. 2017) and the AAs of the constitutive and the MeJA‐triggered defence. We confirmed that JAZs gene expressions were highly correlated with the MeJA‐triggered defence but not with the constitutive defence, suggesting that the latter may act via a JA‐independent pathway (Figure 5C).

### 2.4 Metabolomic Analysis of Seed Exudates to Identify Potential Antimicrobial Molecules

**FIGURE 6:**
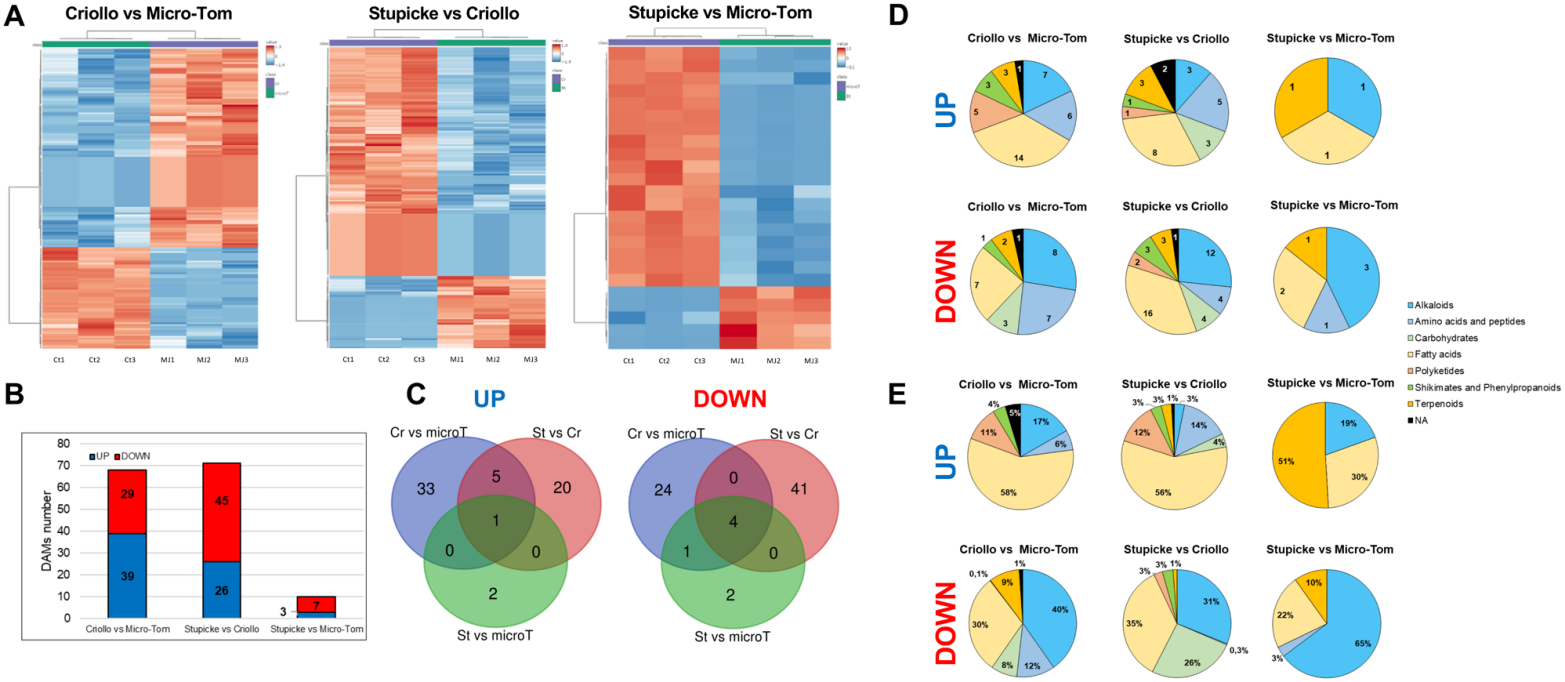
Metabolomic analysis of genotype effect on primed seed exudates. (A) Heatmaps with hierarchical clustering showing an overview of changes in abundance of detected metabolic features in the exudates harvested from Micro‐Tom, Stupicke and Criollo seeds. Colour scales represent log_2_ fold change values. Ct, mock‐primed samples; MJ, methyl jasmonate (MeJA)‐primed samples. (B) Histogram summarising the number of detected differentially accumulated metabolites (DAMs) in the exudate samples. Significant differences (control versus MeJA) were detected with a Student's *t* test coupled with a Bonferroni test to control false discovery rate. (C) Venn diagrams showing the distribution of up‐ and downregulated DAMs across the three genotypes. Cr, Criollo; microT, Micro‐Tom; St, Stupicke. (D) Pie charts showing the distribution of up‐ and downregulated DAMs for the three genotypes comparisons across metabolic pathways in respect to their number. (E) Pie charts showing the distribution of up‐ and downregulated DAMs for the three genotypes comparisons across metabolic pathways in respect to their relative abundance. NA, not annotated. Only features that were validated with the Sirius 4 algorithm (Dührkop et al. 2019, 2021) were classified as DAMs.

To further characterise exudate composition and elucidate the observed AA, we performed untargeted metabolomic analysis (LC–MS/MS) of seed exudates, taking into account genotype and MeJA‐priming factors (Figure 6). Considering all three genotypes, we were able to identify 568 differentially accumulated metabolites (DAMs) between MeJA‐treated and mock‐treated samples. Regarding the genotype effect, pairwise comparisons using exclusively control (non‐primed) samples were performed, positioning the genotype with the strongest AA first: Criollo versus Micro‐Tom, Stupicke versus Criollo, Stupicke versus Micro‐Tom. This led to the detection of 68 DAMs for the Criollo versus Micro‐Tom comparison, 71 DAMs for Stupicke versus Criollo and 10 DAMs for Stupicke versus Micro‐Tom (Figure 6B). Venn diagrams highlighted that one upregulated DAM and 4 downregulated DAMs were in common between all three comparisons (Figure 6C). The upregulated DAM is annotated as [4‐(aminomethyl)phenyl] methanamine, and it is classified as an alkaloid. The downregulated DAMs were annotated as two alkaloids, one terpenoid and one fatty acid. We then analysed their distribution in function of their biochemical nature, in terms of absolute DAM number and relative abundance. Pie charts in Figure 6D showed biochemical families for DAMs with increased and decreased accumulation in respect to the three pairwise comparisons. Overall, an increase in the number of alkaloids in downregulated DAMs for all three comparisons was noticed, passing from 7, 3 and 1 to 8, 12 and 3 in the three comparisons, respectively (Figure 6D). For the relative quantification, upregulated pie charts were mostly dominated by fatty acids while downregulated ones were characterised by a prevalence of alkaloids (Figure 6E).

**FIGURE 7:**
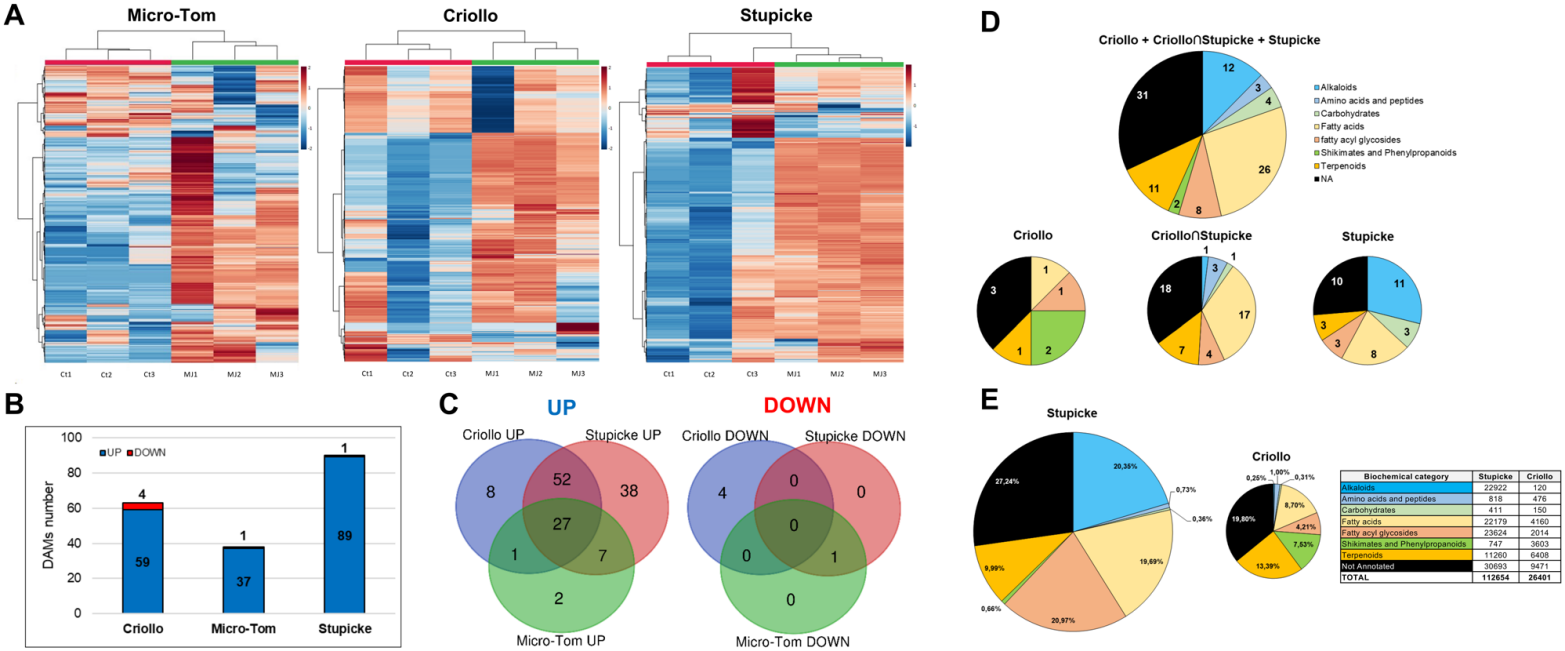
Metabolomic analysis of methyl jasmonate (MeJA) priming effect on seed exudates. (A) Heatmaps with hierarchical clustering showing an overview of changes in abundance of detected metabolic features by comparing the three genotypes Criollo, Micro‐Tom, and Stupicke between them. Colour scales represent log_2_ fold change values. Ct, mock‐primed samples; MJ, MeJA‐primed samples. (B) Histogram summarising the number of detected differentially accumulated metabolites (DAMs) in the exudate samples in the three comparisons. Significant differences (control vs. MeJA) were detected with a Student's *t* test coupled with a Bonferroni test for false discovery rate (FDR) control. (C) Venn diagrams showing DAMs between the comparisons. (D) Pie charts showing the distribution of upregulated DAMs in Stupicke and Criollo genotypes across metabolic pathways in respect to their number. The upper chart represents all the upregulated DAMs specific to Stupicke and Criollo, while the lower charts represent Stupicke‐ and Criollo‐specific DAMs (Stupicke, Criollo) and DAMs in common between them (Stupicke∩Criollo). (E) Pie charts and table showing the distribution of upregulated DAMs in Stupicke and Criollo genotypes across metabolic pathways in respect to relative abundance. The area of the pie charts is proportional to the total abundance of DAMs of the two genotypes. DAMs were quantified in relative units. Only features that were validated with the Sirius 4 algorithm (Dührkop et al. 2019, 2021) were classified as DAMs.

Next, we analysed the MeJA priming effect on the metabolome of seed exudates (Figure 7). When considering all cultivars together, 135 DAMs showed significantly higher accumulation and 5 DAMs showed significantly lower accumulation in the exudates of MeJA‐treated seeds compared to controls (Table S4). Regarding the genotype‐specific responses, MeJA had the strongest impact on Stupicke seed exudates (Figure 7A,B). Overall, a good discrimination between control and MeJA‐treated samples was observed for all three cultivars (Figure 7A). Among the detected DAMs, 2 were specific to Micro‐Tom, 12 to Criollo and 38 to Stupicke (Figure 7C). Venn diagrams highlighted 52 common DAMs between Criollo and Stupicke, while only one common DAM was found between Micro‐Tom and the other two cultivars (Figure 7C). By following the same approach used for transcriptomic data, we focused on the DAMs showing higher accumulation in Criollo and/or Stupicke compared to the other varieties. A high number of DAMs specific to Criollo and/or Stupicke belong to the fatty acids and glycosides category (32 DAMs out of 97), followed by alkaloids (12) and terpenoids (11, Figure 7D). Relative quantification by biochemical category showed that lipids and fatty acids constitute the most represented category for MeJA‐treated Criollo and Micro‐Tom exudates, while in Stupicke, alkaloids were the most represented category (Figure 7E). Taking together all the 135 DAMs with increased accumulation from all genotypes combined, fatty acids emerged as the main biochemical category (Figure S2).

### 2.5 The Proteinaceous Fraction of the Exudates Plays a Major Role in Determining the Antimicrobial Activity

**FIGURE 8:**
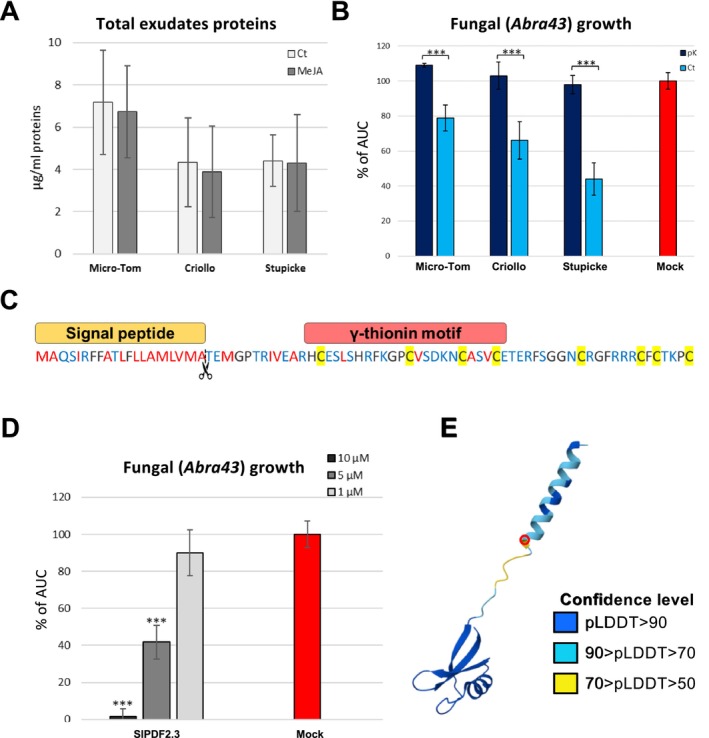
Role of proteins in tomato seed defences against *Alternaria brassicicola*. (A) Histogram representing total protein concentrations in exudates as determined by Bradford assay, *n* = 3. Error bars represent SD. (B) Histogram representing growth inhibition of *A. brassicicola* in presence of exudates of Micro‐Tom, Criollo and Stupicke treated or not with thermolabile proteinase K. pK, exudates treated with proteinase K; Ct, untreated exudates. Values are expressed as percentage of the total area under the curve (AUC) compared to the untreated control. Asterisks indicate significant differences according to Student's *t* test (***p < 0.001). Error bars represent SD. (C) Overview of the amino acid sequence of the putative defensin peptide SlPDF2.3 with predicted signal peptide and conserved motif. Hydrophobic residues are written in red and hydrophilic residues in blue. Cysteine residues are highlighted in yellow. The scissor icon with the dotted line indicates the predicted cleavage site (probability 0.92 according SignalP v. 6.0). Motif analysis was performed with the ScanProsite tool (prosite.expasy.org). (D) Histogram representing growth inhibition of *A. brassicicola* Abra43 in the presence of SlPDF2.3 at different concentrations. Values are expressed as percentage of the total AUC compared to the untreated control. Asterisks indicate significant differences according to Student's *t* test (***p < 0.001). Error bars represent SD. (E) Molecular model of SlPDF2.3 structure made with the AlphaFold3 server. Colour code legend indicates confidence level as predicted local distance difference test (pLDDT) score. Red circle indicates the predicted cleavage site.

After characterising seed exudate metabolites, we focused on the proteinaceous fraction. A first quantification of the total exudates' protein content through the Bradford assay revealed relatively low total protein quantities, ranging between 7.18 (Micro‐Tom mock) and 3.89 μg/mL (Criollo MeJA) (Figure 8A). No significant effect of MeJA treatment was detected in all three genotypes. We then attempted to understand whether the detected proteinaceous fraction contributes to the observed AA by treating the exudates with thermolabile proteinase K, a broad‐spectrum serine protease. We selected a thermolabile form to minimise the effect of the heat treatment on other exudate components such as metabolites (Houlihan et al. 2019). Data obtained showed that the totality of the AA of the exudates was lost using the proteinase K (Figure 8B), indicating that the integrity of the exudates' proteinaceous fraction is required for the AA, either due to the direct antimicrobial effects of proteins or due to modifying enzymes conferring an antimicrobial activity to metabolites. To discriminate between these individual or combinatorial strategies, we performed a proteomic analysis on seed exudates obtained from different genotypes with or without MeJA treatment using mass spectrometry to identify exudate protein composition. However, the seed osmopriming treatment using polyethylene glycol (PEG) prior to exudate collection masked most of the spectrum, rendering the dataset incomplete despite decontamination efforts. Therefore, we took advantage of the transcriptomic dataset to seek proteins/peptide candidates with direct antimicrobial effects. Nevertheless, exploitable proteomic data obtained from MeJA‐treated samples of the three genotypes pooled indicated the presence of three defensins (Solyc07g007750, Solyc07g007710 and Solyc04g072470) and several other defence‐related proteins, such as PR proteins and proteases in the exudates, despite the impossibility of quantifying them (Table S5).

### 2.6 A Defensin Induced by MeJA in Seeds Showed Antifungal Activity Against *A. brassicicola*

Based on the indications of the proteinase K assay and the transcriptomic analysis results, and in order to functionally characterise candidates, we looked at the expression of genes encoding peptides with potential antimicrobial activity, induced by MeJA in the three genotypes and correlated to the AA against Abra43. Out of the 285 genes induced by MeJA in all three genotypes, the expressions of many genes were highly correlated with MeJA accumulation and AA (Table S2). Among them, our attention was caught by *Solyc07g007755*, whose expression was highly correlated with the antimicrobial activity (*r* = 0.83; adjusted *p*‐values of 0.004 for Criollo, 8.6e^−7^ for Micro‐Tom, 0.00015 for Stupicke; log_2_ fold change values of 1.4 for Criollo, 1.58 for Micro‐Tom, 1.99 for Stupicke). *Solyc07g007755* encodes a 78 amino acid peptide annotated as a defensin‐like protein precursor showing sequence similarity with *Arabidopsis thaliana* PDF2.3 (63.6% of protein sequence identity), a well‐characterised defensin with antifungal activity (Vriens et al. 2016). The tomato full‐length amino acid sequence contained a signal peptide that addresses it to the secretory pathway according to SignalP v. 6.0 (https://services.healthtech.dtu.dk/services/SignalP‐6.0/) and is likely absent in the mature protein (Figure 8C). Structural modelling was based on the P322 defensin from potato ( *Solanum tuberosum* ) (Stiekema et al. 1988), resulting in a very high overall confidence of the model (GMQE value of 0.92) (Figure 8E). The expression of the *Solyc07g007755* gene has been reported to be upregulated following interaction with the beneficial fungus *Trichoderma harzanium* (Aamir et al. 2023). To investigate its potential role in seed defence responses, we obtained a synthetic Solyc07g007755 peptide using the 58 amino acid sequence of the putative mature peptide and added it at different concentrations to Abra43 liquid culture. We favoured this approach over genetic manipulation due to defensins family functional redundancy. This assay indicated that the presence of Solyc07g007755 peptide was able to inhibit almost completely Abra43 growth at 10 μM, while the inhibition was not significant anymore at 1 μM (Figure 8D). These data support the hypothesis that this putative defensin contributes to the arsenal of seeds' defence mechanisms against fungal pathogens at the germination stage. We renamed the peptide SlPDF2.3, in reference to its *A. thaliana* closest homologue.

## 3 Discussion

Despite their primary importance in the food security question, seed–pathogen interactions are poorly characterised to date. It is likely that the notion of seeds as passive pathogen carriers hampered the development of this research field. However, a novel research impulse is making this vision obsolete, suggesting the existence of a dynamic dialogue (Ortega‐Cuadros et al. 2022; Darrasse et al. 2024). Our work fits into this framework, indicating that defence pathways can be elicited already at the seed stage. This conclusion was made possible by circumventing the limitation of establishing a seed‐based pathosystem through the exploitation of seed exudates in liquid cultures (Hubert, Leprince, et al. 2024).

Several defence strategies were previously described in seeds, depending on the species and ecological niche (Dalling et al. 2020). Seed dormancy, a physiological state that prevents seeds from germinating in unfavourable conditions, is known to be linked with the presence of constitutive defences of different nature (Dalling et al. 2011; Hubert, Marchi, et al. 2024). Seed dormancy can be largely explained by physical and physiological dormancies, depending on the nature of the germination inhibition mechanism (Finch‐Savage and Leubner‐Metzger 2006). In the case of physical dormancy, physical defences based on the seed coat are prevalent, resulting in decreased seed permeability to microbial colonisation, while physiological dormancy could mostly be associated with biochemical defences such as the activity of antimicrobial enzymes in the spermosphere (Dalling et al. 2011; Pollard 2018). Indeed, several studies have already shown that seeds can exude molecules with AA of different natures, such as specialised metabolites, enzymes and peptides (Rocha et al. 2015; Bednarz et al. 2019; Houlihan et al. 2019). The main novelty of the present study in relation to the state of the art is the demonstration that the AA of seed exudates can be inducible. To date, elicitor‐dependent defence induction has been shown only on fully germinated seedlings, while available studies about seed exudates did not investigate the induction (or enhancement) of the AA (Shailasree et al. 2001; Worrall et al. 2012; Rocha et al. 2015; Song et al. 2017; Houlihan et al. 2019). On one hand, this provided evidence for a previously unsuspected dynamism of seed defence mechanisms, while on the other hand, it generated a dataset of genes and metabolites associated with constitutive and inducible defences, holding potential for the development of novel phytosanitary strategies to apply at the seed stage. While direct seed defence priming with phytohormones negatively affected germination (Figure S1), we cannot exclude that other types of elicitors might trigger protection without compromising germination.

Regarding the effectiveness of the induced seed defence pathways, results summarised in Figure 2B suggested a limited inhibitory effect on the growth of compatible pathogens of both bacterial and fungal nature and an overall scarce, if not stimulatory, effect on bacterial pathogens in general. Damping‐off pathotests with the compatible oomycete pathogen *Pythium ultimum* further consolidated this finding (Figure S3). This is consistent with recent research showing antimicrobial properties of tomato and maize seed exudates towards non‐compatible pathogens belonging to fungal (*A. brassicicola*) and oomycete (*Phytophthora sojae*) classes (Zhang et al. 2022; Hubert, Leprince, et al. 2024). Why do the tested exudates have no or little inhibitory effect on compatible pathogens? A possible explanation might be that compatible, host‐specialised pathogens have evolved detoxification mechanisms against broad‐spectrum defence compounds that might therefore represent a first nonspecific layer of defence against a cohort of potentially pathogenic soil microbes. A larger panel of pathogens would be required to further challenge such a hypothesis. While seed exudates showed a strong inhibitory effect against fungi, no activity was observed towards bacterial pathogens. This conclusion might be just due to specific cases. However, it is worth noting that seed infestations by fungal pathogens can result in direct damage to seed prior to germination (e.g., seed rot), while bacterial colonisation is typically symptomless, with the pathogen behaving as a commensal endophyte (Darrasse et al. 2010; Dutta et al. 2014; Pedraza et al. 2018; Larran et al. 2020). This is exemplified by the recent ‘ceasefire’ model proposed for the bean seed– *Xanthomonas citri* pv. *fuscans* interaction, where it is suggested that both host and bacterial pathogen prefer to ‘prepare to battle’ later after germination rather than getting engaged at the seed imbibition or early germination stage (Darrasse et al. 2024). The fact that *Alternaria* fungi can behave as saprotrophs (Taj et al. 2015) might further suggest that seed defence would be strategically fungi‐oriented, especially during the critical germination stage (Ortega‐Cuadros et al. 2022).

Characterisation of the transcriptome of imbibed seeds resulted in more than 10,000 DEGs for each genotype comparison, indicating remarkable intraspecific variability (Figure 3). This suggested that the differences in constitutive AA between the genotypes might be due to different molecular mechanisms, rather than a different degree of activation of a similar mechanism. The analysis illustrated in Figure 3B revealed that the three TF families FAR1, MYB and NF‐YA are interesting candidates to play prominent roles in mounting constitutive defences (Figure 3C). Despite the large size and functional diversity, members of the NF‐YA, MYB and FAR1/FHY3 TF families are known to play a role during seed development in *A. thaliana* (Mu et al. 2013; Ma and Li 2018) but also in plant defence as regulators of the JA signalling in adult plants (Zhang et al. 2022; Sun et al. 2015; Liu et al. 2019; Tan et al. 2022). However, we could not find any information about the involvement of detected genes either in defence processes or in seed germination (Figure 4C). In respect of the tested elicitors, classic phytohormones SA (represented by Bion) and MeJA differ greatly in terms of capacity to induce AA in exudates (Figure 2B). The classical scheme predicts that JA‐dependent pathways are effective against necrotrophs, while SA‐dependent pathways act against biotrophs, although there are exceptions to this axiom (Glazebrook 2005; Pieterse et al. 2012). Therefore, at first glance, one can think that the effect of MeJA on the necrotrophic fungi *A. alternata* and Abra43 could be expected, while in the case of Bion, the lack of inhibitory effect on bacteria (biotrophs) is more puzzling. However, the necrotroph/biotroph dichotomy might not hold true at the seed stage, where most of these pathogens persist as symptomless endophytes until germination or seedling establishment, when they switch to pathogenicity (Pochon et al. 2013; Chen, Ruh, et al. 2021). Consistently, a marked switch in defence pathways activation before and after germination was recently observed in the *A. brassicicola–A. thaliana* pathosystem (Ortega‐Cuadros et al. 2022).

Nevertheless, it remains evident that MeJA treatments induced transcriptional activation of several genes associated with defence pathways, such as WRKY TFs and ERF TFs (Pre et al. 2008; Wang et al. 2022) (Figure 4, Table S2) at the expense of germination vigour (Figure S1). The 16 MeJA‐upregulated genes in common between Stupicke and Criollo genotypes (Figure 4D) are interesting candidates to explain the MeJA‐induced AA. Out of them, two (*SlEIN3* and *SlETR4*) are known to be involved in the ethylene‐response pathway (Kevany et al. 2007; Yang et al. 2010), one *bHLH* (*SlbHLH13*) is known to negatively regulate JA responses (Sasaki‐Sekimoto et al. 2013), and one *MYB* was closely related to a MYB TF (MYB108) known in *A. thaliana* to regulate cuticle‐related immunity to *Botrytis cinerea* and in *Brassica rapa* to promote Verticillium wilt resistance (Cui et al. 2022; Su et al. 2023). Five WRKY (*SlWRKY16*, *SlWRKY37*, *SlWRKY39*, *SlWRKY75*, *SlWRKY78*) and two putative MYB (*SlMYB13*, *SlGAMYB‐like2*) TF genes were also present in the MeJA‐induced 285 genes list (Table S2) and are well‐known constituents of JA‐dependent pathways activated in response to necrotrophic pathogens in adult plants (Zhang 2015; Ren et al. 2020; Chen, Zhang, et al. 2021). These data suggested that MeJA treatment activates both JA and ethylene pathways in seeds. JA signalling appears to play a multilayered role in seed defence, as transcriptomics data suggest that JA might be rather negatively correlated with constitutive defences (Figure 5A). This appears plausible considering the crosstalk between JA and abscisic acid, delaying seed germination (Figure S1) (Varshney and Majee 2021). On the other hand, SA signalling appears to not play a defensive role against the tested pathogens, excluding a weak, but significant, 17% reduction of *A. alternata* growth with Criollo exudates (Figure 2B). Instead, Bion priming led to several cases of increased microbial growth, suggesting an overall negative role for SA in seed defence induction.

Besides TFs, genes encoding proteinases and proteinase inhibitors were strongly induced by MeJA (Table S2), suggesting that they might play a role in the enzymatic battle between seeds and pathogens, targeting pathogen enzymes and effectors (Pollard 2018). It is also interesting to note the presence of an oxidoreductases cluster in the MeJA‐upregulated categories (Figure 4C). Previous studies have already shown the presence of polyphenol oxidase and peroxidase enzymatic activities within the outer seed layers of cereal species such as wild oat ( *Avena fatua* ) and wheat ( *Triticum aestivum* ) (Jerkovic et al. 2010; Fuerst et al. 2014). Finally, by looking at the gene expression dataset in correlation with AA against Abra43, we noticed several proteins with putative roles in defence, including proteinases, proteinase inhibitors, chitinases, PR proteins and defensins (Table S2). This suggests a certain extent of similarity with defences of vegetative tissues, although functional validation would be required to test this hypothesis.

Complementing the transcriptomic approach, we generated for the first time a comprehensive overview of the metabolome of tomato seed exudates (Figures 6 and 7). Although our data did not link them to Abra43 growth inhibition, specialised metabolites have well‐known defence properties against many pathogens. Globally, DAM profiles for the genotype effect were dominated by fatty acids and alkaloids (Figure 6D,E). Such a marked presence of fatty acids is unexpected and triggers questions about their origin and properties. To the best of our knowledge, transport and exudation of fatty acids from inner seed tissues have never been reported. Leakage from the seed coat might be an alternative, although it sounds unlikely because of the highly hydrophobic nature of the polymers constituting the outer coat layers. We cannot exclude those metabolites leaked from the inner tissues as radicles broke the seed coat during germination. Regardless of their origin, our data indicate substantial genotypic differences among the identified fatty acid pools (Figure 6D,E). Regarding alkaloids, their presence in tomato seeds has already been reported (Kuang et al. 2020; Takeda et al. 2021).

MeJA treatment triggered an extensive reshaping of the seed exudates metabolome (Figure 7). Interestingly, fatty acids/acyl glycosides were globally the most represented family in the upregulated DAMs of the two MeJA‐responsive genotypes (Stupicke and Criollo), accounting for 40.6% in Stupicke, the genotype with the highest AA (Figure 7D,E). This suggests that MeJA treatment heavily impacts fatty acid metabolism and transport in tomato seeds. Another observation is the enrichment of alkaloids in Stupicke's exudates in response to MeJA (Figure 7D,E). It remains to be verified whether these metabolites are functional against other pathogens or in the context of soil multitrophic interactions. A plethora of compounds belonging to this class are known to possess antimicrobial properties (Zhang et al. 2009; Sulaiman et al. 2022).

The pK assay indicated that the antimicrobial properties of the identified metabolites are negligible against Abra43 (Figure 8), although we cannot totally exclude that the 55°C treatment used to stop the assay could have degraded some thermolabile metabolites such as small terpenoids (de Matos et al. 2019). Another hypothesis is that these metabolites may acquire antimicrobial properties following specific decorations due to enzymes present in the exudates. Interestingly, we observed a correlation between the AA following MeJA treatment and the expression of several genes encoding putative enzymes such as glycosyltransferase (*Solyc06g072880.2.1*, PCC = 0.95), acyltransferase (*Solyc02g093180.3.1*, PCC = 0.87) and several hydroxylases (*Solyc02g090350.4.1*, PCC = 0.85; *Solyc10g007960.1.1*, PCC = 0.98; *Solyc04g078325.1.1*, PCC = 0.86). However, taken together with previous studies, it appears that seed exudates exert AA mostly via the action of proteins and peptides (Rose et al. 2006; Rocha et al. 2015; Raviv et al. 2017; Houlihan et al. 2019). Consistently, we were able to functionally validate a defensin gene correlated with AA against Abra43, *SlPDF2*.3 (PCC = 0.83) (Figure 8D). Although we failed to produce direct evidence of its presence in the exudates, our data show that defensins can be secreted in the spermosphere (Table S5). Two out of the three detected defensins are transcriptionally induced by MeJA: *Solyc07g007710* in Criollo, *Solyc04g072470* in Micro‐Tom (Table S2), with *Solyc07g007750* and *Solyc07g007710* belonging to the same genomic cluster of *Solyc07g007755*/*SlPDF2.3*, formed by six defensin genes. This dataset further showed the presence of several heat shock proteins, redox proteins and other defence‐related proteins such as proteases and pathogenesis‐related (PR) proteins (Table S5). The PEG contamination issue highlights the need to develop a novel sample preparation strategy. That said, the apparent convergence between seed transcriptomic and exudates proteomic data regarding categories such as defensins, proteases/protease inhibitors and redox enzymes suggests that they might form a defensive cocktail against pathogens, making them prominent candidates to further investigate defence induction in seeds.

To summarise, the present study provides novel leads to stimulate research on seed–pathogen interactions and adds new details to the current understanding of compatible/incompatible interactions mechanisms. Although we could not observe an AA on compatible pathogens, our data identify novel candidate antimicrobial molecules that can be exploited in regard of other crops, such as rapeseed ( *Brassica napus* ) or cabbage ( *Brassica oleracea* ) in the case of *A. brassicicola*.

## 4 Experimental Procedures

### 4.1 Seed Material

*Solanum lycopersicum* ‘Stupicke’, ‘Criollo’ and ‘Micro‐Tom’ were used in this study. Plants were grown under semi‐controlled greenhouse conditions at the INRAE site of Angers (France) between November and July 2021 in 15 L pots (containing Klassman‐Deilmann substrate 5) watered with a nutrient solution and supplemented with 16 h of 250 μmol m^−2^ s^−1^ of light. The day/night temperatures were set up at 23°C/20°C (daily mean and maximal temperatures of 21.2°C and 28.8°C), respectively. Mature red fruits were then harvested from the 2nd to the 8th truss. Fruits were then cut to extract seeds by incubating the locular tissues for 1 h under gentle shaking at room temperature (RT) in 100 mL Erlenmeyer flasks containing 40 mg of pectolytic enzymes (Lafazym CL). Seeds were thoroughly washed with tap water to remove fruit tissue debris. Seeds were then dried in a desiccator under a controlled airflow at 43% relative humidity (RH) and at RT for 48 h before being stored at 4°C in the dark.

### 4.2 Seed Priming and Seed Exudates Production

Seed osmopriming solutions were prepared as follows: 28.5 g polyethylene glycol (PEG) 8000 was added to 100 mL Milli‐Q sterile water to generate a solution with osmotic potential of −1 MPa at RT. To prepare solutions for elicitor treatments, the following elicitor final concentrations were used: 10 mM β‐aminobutyric acid (BABA, Sigma‐Aldrich); 2 mM MeJA (Sigma‐Aldrich); 10 mM for the active compound of Bion 50 WG (granular form composed of 50% by acibenzolar‐*S*‐methyl, Syngenta). The concentrations used were comparable to those used in similar studies on tomato (Worrall et al. 2012; Smart et al. 2013). 2 mL of seed priming solution (1 MPa ± elicitors) was then added into 30 mm diameter sterile glass Petri dishes containing 30 dry mature seeds on sterile Whatman filter paper to imbibe in the dark at 20°C for 48 h. Three biological replicates of 30 seeds each were used for each treatment. Seeds were then abundantly washed under tap water to remove the priming solution, blot‐dried and placed into a desiccator at 43% RH for 48 h to redry them. After complete drying, seeds were placed again in 30 mm diameter sterile glass Petri dishes and imbibed using 2 mL sterile Milli‐Q water for 96 h in the dark at 20°C to produce exudates (Hubert, Leprince, et al. 2024). Exudates were collected with sterile tips and filter‐sterilised with CHROMAFIL Xtra PA‐20/13 0.20 μm filters (Macherey‐Nagel GmbH & Co.), aliquoted in sterile 2 mL Eppendorf tubes and flash‐frozen in liquid nitrogen. Exudates were stored at −80°C before use. The whole procedure is summarised in Figure 1.

### 4.3 Microbial Material

*Alternaria brassicicola* Abra43 (Pochon et al. 2012) and *A. alternata* NB100 (Bessadat et al. 2014) were maintained at 25°C on potato dextrose agar (PDA) and potato carrot agar plates, respectively. Inoculum was prepared as previously described (Hubert, Leprince, et al. 2024). A final inoculum concentration of 10^3^ conidia/mL was used for experiments. *Clavibacter michiganensis* subsp. *michiganensis* (Cmm) rifampicin‐resistant strain Cm 144 (CFBP 7572) was maintained on yeast extract‐dextrose‐CaCO_3_ (YDC) plates at 25°C, while *Xanthomonas campestris* pv. *campestris* (Xcc) rifampicin‐resistant strain ATCC 33913 (Darrasse et al. 2010) was maintained on tryptic soy agar (TSA) plates at 28°C. Both media were supplemented with 50 μg/mL rifampicin. All nutrient media mentioned were obtained from Duchefa Biochemie. For inoculum preparation of bacteria, 5 mL tryptic soy broth (TSB) liquid cultures were incubated overnight at 28°C and 150 rpm; then the inoculum was diluted to an OD_600_ value of 0.15 for Cmm and 0.10 for Xcc, corresponding to 10^8^ CFU/mL. A final CFU concentration of 10^5^ was used for experiments. Pure culture of *Pythium ultimum* DV1505‐941 was routinely cultured on potato dextrose agar (PDA, BIOKAR Diagnostics) in the dark at 22°C.

### 4.4 Fungal and Bacteria Growth Curves

To follow fungal growth, we performed nephelometry assays. Both Abra43 and NB100 *A. alternata* inocula were added to 96‐well transparent flat‐bottom plates (Greiner) containing potato dextrose broth (PDB) and water or seed exudates in the following proportions: 8/10 of PDB, 1/10 of inoculum (concentration of 10^4^ conidia/mL, for a final concentration of 10^3^), 1/10 of exudates (treatment) or Milli‐Q sterile water (control), for a final volume of 300 μL for each mix. Fungal growth was then measured with a nephelometric reader at 635 nm (NEPHELOstar Galaxy, BMG Labtech) (Joubert et al. 2010) at 20°C, 500 rpm and for 66 h and 20 min. Growth data were analysed using the Omega MARS software v. 3.42 R5 (BMG Labtech) as previously described (Hubert, Leprince, et al. 2024).

To follow bacterial growth, we performed spectrometry assays (Kurokawa and Ying 2017). Rifampicin‐resistant Cmm and Xcc inocula were added to 96‐well transparent flat‐bottom plates (Greiner) containing nutrient broth yeast (NBY) for Cmm and TSB for Xcc and water or exudates in the following proportions: 8/10 of NBY or TSB, 1/10 of inoculum (concentration of 10^6^ CFU/mL, for a final concentration of 10^5^), 1/10 of exudates (treatments) or of Milli‐Q sterile water (control) for a final volume of 300 μL. All bacterial growth media were supplemented with 50 μg/mL rifampicin. Bacterial growth was then measured with a spectrophotometric reader at 600 nm (SPECTROstar, BMG Labtech) at 25°C for Cmm and 28°C for Xcc, 500 rpm and for 96 h. Growth data were analysed using the Omega MARS software v. 3.42 R5 (BMG Labtech).

### 4.5 Statistical Analysis of Growth Curves

Data corresponding to fungal and bacterial growth curves data were exported from the Omega MARS software and analysed in R studio (v. 4.3.2) as described in Hubert, Leprince, and Buitink (2024). Values of the area under the curve (AUC) were used to test the effects of exudates on microbial growth, and growth ratio values calculated from the AUC of the pathogen's growth with and without the presence of exudate (% growth = AUC (condition)/AUC (control) × 100). Data normality was tested using the Shapiro–Wilk test. Significance between pairwise comparisons was tested with a Student's *t* test (*p* < 0.05), while a Mann–Whitney test was used (*p* < 0.05) in case of failure of the normality test. For multiple comparisons, an ANOVA test (*p* < 0.05) was performed. In case the normality test failed, a Kruskal–Wallis test (*p* < 0.05) followed by Dunn's post hoc test (*p* < 0.05) was used instead. Respective tests used for nephelometry/spectrometry results are indicated in figure legends.

### 4.6 *Pythium ultimum* Pathotest and Statistics

*Pythium ultimum* was cultured for 14 days on V8 agar (3 g CaCO_3_, 20 g agar, 200 mL V8 vegetable juice, Continental Foods Europe) with cellophane films (10 × 10 cm). The mycelium was removed from the plates and mixed until homogeneity with phosphate‐buffered saline (PBS, pH 6.5). Twelve Petri dishes were used for one tray of 40 wells. The mycelium mixture was added to fertilised water (1 g/L of PLANT‐PROD 15–10‐30) and mixed with soil (TRAYSUBSTRAT 75/25—RECETTE 092, Klasmann‐Deilmann). The potting soil was watered with fertilised water until saturation. In order to maintain the soil moisture, twice a week watering was made with 300 mL of fertilised water. After the saturated soil was evenly distributed over the trays (1 seed/tray), trays were then placed in a culture chamber (23°C/20°C; 16/8 h; 80% RH). Fourteen days after sowing, the plant emergence status was determined according to the following criteria: no germination (0), plant began to germinate (1), seedling (2), plant had visible signs of infection (sick). Seedling emergence was analysed with logistic generalised linear models, and confidence interval values of 95% were extracted. The log odd values predicted by the model were converted to infection probabilities, and 95% confidence intervals were calculated and displayed as error bars (Figure S3).

### 4.7 Transcriptomic Analysis

RNA samples were extracted from the same seed lot used to produce exudates following the seed priming treatment with or without elicitors using the NucleoSpin RNA Plant and Fungi kit (Macherey‐Nagel) with lysis buffer containing 1% polyvinylpyrrolidone (PVP‐40) followed by incubation at RT for 10 min. RNA quantity and quality were measured using a NanoDrop ND‐1000 (NanoDrop Technologies) and a 2100 Bioanalyzer (Agilent Technologies), respectively. All samples showing A_260_/A_280_ and A_260_/A_230_ ratio > 1.8, RNA Integrity Number, RIN > 7 and 28S/18S > 1.7 were sent to Beijing Genomics Institute (https://www.bgi.com, Hong Kong, China) for library preparation and sequencing on BGISEQ‐500 platform, generating a minimum of 20 million reads of 100 bp per sample (20 M PE100). After adapter trimming and quality control, high quality reads were mapped on the tomato reference transcriptome based on SL4.0 genome version and ITAG4.0 annotation (Hosmani et al. 2019) using quasi‐mapping alignment and quantification methods of Salmon algorithm v. 1.2 (Patro et al. 2017). For gene expression analysis, raw RNA‐Seq counts were first normalised as transcripts per kilobase million (TPM). To identify DEGs, the R package SARTools (Varet et al. 2016) including DEseq2 algorithm (Love et al. 2014) was used and an adjusted *p*‐value threshold of 5% was set to identify DEGs following the MeJA treatments. ClusterProfiler algorithm (Yu et al. 2012) based on GO annotations available from Tomato ITAG4.0 was performed and an adjusted *p*‐value threshold of 5% was set to determine over‐represented functional classes (Lohse et al. 2014).

### 4.8 Untargeted Metabolomic Analysis (LC–MS/MS)

Untargeted metabolomic analyses were performed following the protocol of Boutet et al. (2022), with modifications. Lyophilised seed exudates were resuspended in 100 μL of a solution of 10% acetonitrile. Apigenin (500 ng) was added to each sample and used as an internal standard (Extrasynthese). The resuspended extracts were filtered with glass microfibre filters (Whatman International Ltd) and placed in HPLC vials. A quality control (QC) made up of 8 μL from each sample was prepared in a separate HPLC vial.

Sample injection and data analysis: Untargeted metabolomic analyses were conducted using an ultra‐high‐performance liquid chromatographic system (UPLC, Dionex UltiMate 3000 RSLC, Thermo Fisher Scientific) coupled with a Q‐ToF mass spectrometer (Maxis Impact, Bruker Daltonics) using the same pipeline and parameters indicated in Boutet et al. (2022). Each sample was injected and analysed in positive and negative ionisation modes (ESI+ and ESI−). Raw metabolomic data (.d) were converted into .xml files using MSConvert software (ProteoWizard package 3.0). ESI+ and ESI− data were analysed using mzMine v. 3.0 software (Schmid et al. 2023) (mzmine.github.io), using the protocol described in Boutet et al. (2022). Metabolic feature intensity was normalised by the internal standard (apigenin) and by the mass of lyophilised exudate for each sample. For metabolite annotation, molecular networks were generated with MetGem software (Olivon et al. 2018; https://metgem.github.io). Molecular networks were generated using a cosine score of 0.85 for ESI+ and 0.65 for ESI−. Annotation of metabolic features was carried out in several consecutive steps: (1) match against the IJPB internal library including standards and putatively annotated features (experimental library [mz and RT]: 99 and 67 features for ESI+ and ESI−, respectively; exact mass library [*m*/*z*]: 637 and 475 features in ESI+ and ESI−, respectively). (2) Metabolomic data were matched against available MS2 spectral libraries (Massbank NA, GNPS Public Spectral Library, NIST14 Tandem, NIH Natural Product and MS‐Dial). (3) Features without annotation proposition were assigned to a metabolic family using information from the molecular network. (4) A putative annotation was proposed for non‐annotated interesting features using Sirius software (Dührkop et al. 2021; https://bio.informatik.uni‐jena.de/software/sirius/) or by checking the MS/MS spectra.

### 4.9 Proteomics Analysis

Seed exudates were filtered on 10 kDa filters (Amicon ultra 10 kD, Merck Millipore). The flowthrough was collected, and pH was adjusted to pH 8 using ammonium bicarbonate. The flowthrough was then reduced with dithiothreitol (DTT) and alkylated with iodoacetamide. Desalting of peptides was carried out using the Phoenix peptide preparation kit (Preomics). LC–MS/MS analyses were performed as previously described (Tarkowski et al. 2024) with some modifications. Separation was shortened from 75 to 45 min, and the column was replaced by a Biozen C18 (2.6 μm Peptide XB‐C18, 250 × 0.075 mm) column from Phenomenex. Protein fragments identification and filtering were done by querying the resulting MS/MS data against *S. lycopersicum* ITAG4 database, using X! Tandem Piledriver (v. 2015.04.01.1) and i2masschroq v. 1.2.9 (Langella et al. 2024). Proteins were retained only if they were identified with one peptide with an *E*‐value lower than 0.01 and a protein *E*‐value lower than 0.00001, resulting in a protein false discovery rate (FDR) of 0% and a peptide‐spectrum match (PSM) FDR lower than 0.36%.

### 4.10 Bradford Assay and Proteinase K Assay

Protein quantification according to the Bradford method (Bradford 1976) was performed by using 50 μL of exudates with 200 μL of Coomassie blue reagent. Protein concentrations were calculated using a standard curve of bovine serum albumin (BSA). For the proteinase K assay, thermolabile proteinase K (New England Biolabs) was added at a final concentration of 10 mg/mL to exudates and incubated at 20°C for 24 h (Houlihan et al. 2019). The proteinase K was then inactivated with a 55°C treatment for 10 min. Nephelometry assays were carried out on these samples as described previously.

### 4.11 Peptide Synthesis and Antimicrobial Activity Assay

The SlPDF2.3 peptide was synthesised by Royobiotech Company (Shanghai, China) with a purity > 90% and dissolved in sterile Milli‐Q water prior to addition to Abra43 liquid culture. Its 58 amino acid sequence (TEMGPTRIVEARHCESLSHRFKGPCVSDKNCASVCETERFSGGNCRGFRRRCFCTKPC) corresponds to the protein encoded by the *Solyc07g007755* gene after removing the signal peptide predicted by SignalP v. 6.0 (Teufel et al. 2022). To validate the AA of the peptide, three final concentrations of the SlPDF2.3 were tested in nephelometry: 10, 5, or 1 μM according to the protocol described previously.

## Author Contributions

**Łukasz P. Tarkowski:** conceptualisation, methodology, investigation, formal analysis, writing – original draft preparation, writing – review and editing. **Benjamin Hubert:** investigation, formal analysis, writing – review and editing. **Muriel Marchi:** investigation, writing – review and editing. **Camille Tranchant:** investigation. **Stéphanie Boutet:** investigation, formal analysis. **Céline Brosse:** investigation. **Mathilde Causse:** resources. **Thierry Balliau:** formal analysis. **Mélisande Blein‐Nicolas:** formal analysis. **Massimiliano Corso:** formal analysis, writing – review and editing. **Sébastien Aubourg:** formal analysis, writing – review and editing. **Jérôme Verdier:** conceptualisation, methodology, formal analysis, writing – original draft preparation, writing – review and editing.

## Conflicts of Interest

The authors declare no conflicts of interest.

## Supporting information


**Figure S1:** Germination assay on tomato seeds primed with methyl jasmonate (MeJA). Three biological replicates of 30 seeds were used to assess the effect of MeJA osmopriming on germination rate of the three genotypes Micro‐Tom, Criollo and Stupicke. Lines of different colours indicate the different MeJA dosages used for the osmopriming: Ct, mock control without MeJA; MeJA 2, MeJA 2 mM; MeJA 20, MeJA 20 mM. Seeds were placed in sterile glass petri dishes with sterile Whatman filter paper imbibed in Milli‐Q sterile water. Germination percentage was assessed at 4 and 8 days. The experiment was repeated 2 times with consistent results. Error bars represent SD.

**Figure S2:** Distribution of differentially accumulated metabolites (DAMs) induced by methyl jasmonate (MeJA) treatment from all genotypes. Left, pie chart showing the distribution of DAMs increasingly accumulated following MeJA treatment from all three genotypes combined across metabolic pathways. Right, table resuming their number and providing the colour key for the pie chart.

**Figure S3:** Response to damping‐off of primed tomato seeds. Seeds of Micro‐Tom, Criollo and Stupicke genotypes primed with polyethylene glycol (PEG) 8000 plus β‐aminobutyric acid (BABA) 10 mM (BABA), MeJA 2 mM (methyl jasmonate) or mock (Ct) were sown in trays containing soil inoculated with *Pythium ultimum* (yellow bars, condition ‘Pythium’) or mock‐inoculated (green bars, condition ‘without Pythium’). Germination rates after 14 days were computed as emergency probabilities. The experiment was repeated three times with consistent results, with *n* = 20. Error bars represent 95% confidence intervals.

**Table S1:** Transcriptomic analysis data in respect of the genotype effect used for Figure 3. Column A lists of upregulated differentially expressed genes (DEGs) resulting from the comparison Stupicke versus Criollo. Column B lists of upregulated DEGs resulting from the comparison Stupicke versus Micro‐Tom. Column C list of genes resulting from the comparison of non‐DEGs of Criollo versus non‐DEGs of Micro‐Tom. Column D list of genes resulting from the intersection of the three comparisons.

**Table S2:** Results of the transcriptomic analysis of seeds in regard of the methyl jasmonate (MeJA) treatment effect. The table show differentially expressed genes (DEGs) for the three genotypes Criollo, Micro‐Tom, Stupicke, the 285 commonly upregulated, and the correlation coefficients between the expression values of the 285 genes and the antimicrobial activity of the exudates.

**Table S3:** Details of gene ontology analysis results of the seven over‐represented functional classes showed in Figure 4C. GeneRatio, number of differentially expressed genes (DEGs) belonging to the functional category over total number of DEGs considered; BgRatio, number of genes included expressed in tomato seeds belonging to the functional category over total number of genes expressed in tomato seeds.

**Table S4:** Lists with details of the differentially accumulated metabolites (DAMs) resulted from the untargeted metabolomic analysis of exudates from the three genotypes Criollo, Micro‐Tom and Stupicke in relation to methyl jasmonate (MeJA) treatment. ND, not detected.

**Table S5:** List of detected peptides and proteins in exudates from methyl jasmonate (MeJA)‐treated seeds following cleanup processing with the Phoenix kit. Heat shock, redox and defence‐related proteins are highlighted in red, yellow and blue, respectively. Asterisks indicate defensins. Annotations are from the ITAG4.0 *Solanum lycopersicum* genome functional annotation version. Exudates represent a pool of the three genotypes Criollo, Micro‐Tom and Stupicke.

## Data Availability

The data that support the findings of this study have been deposited in NCBI Gene Expression Omnibus and are publicly accessible through GEO accession number GSE277291. The metabolomic data and metadata have been deposited at the MassiVE data repository portal with the identifier MSV000096553 (doi: 10.25345/C54T6FF8C).
