# Supplementary material for: Antimicrobial Peptides as Part of the Arsenal of Constitutive and Inducible Seed Defences in Tomato Seed Exudates Against Pathogens
Source: Mol Plant Pathol. 2025 Oct 27;26(10):e70164. doi: 10.1111/mpp.70164 (PMC12558808; doi:10.1111/mpp.70164)
Supplement: Supplementary file 1 — Figure S1: Germination assay on tomato seeds primed with methyl jasmonate (MeJA). Three biological replicates of 30 seeds were used to assess the effect of MeJA osmopriming on germination rate of the three genotypes Micro‐Tom, Criollo and Stupicke. Lines of different colours indicate the different MeJA dosages used for the osmopriming: Ct, mock control without MeJA; MeJA 2, MeJA 2 mM; MeJA 20, MeJA 20 mM. Seeds were placed in sterile glass petri dishes with sterile Whatman filter paper imbibed in Milli‐Q sterile water. Germination percentage was assessed at 4 and 8 days. The experiment was repeated 2 times with consistent results. Error bars represent SD. [file MPP-26-e70164-s007.pdf]

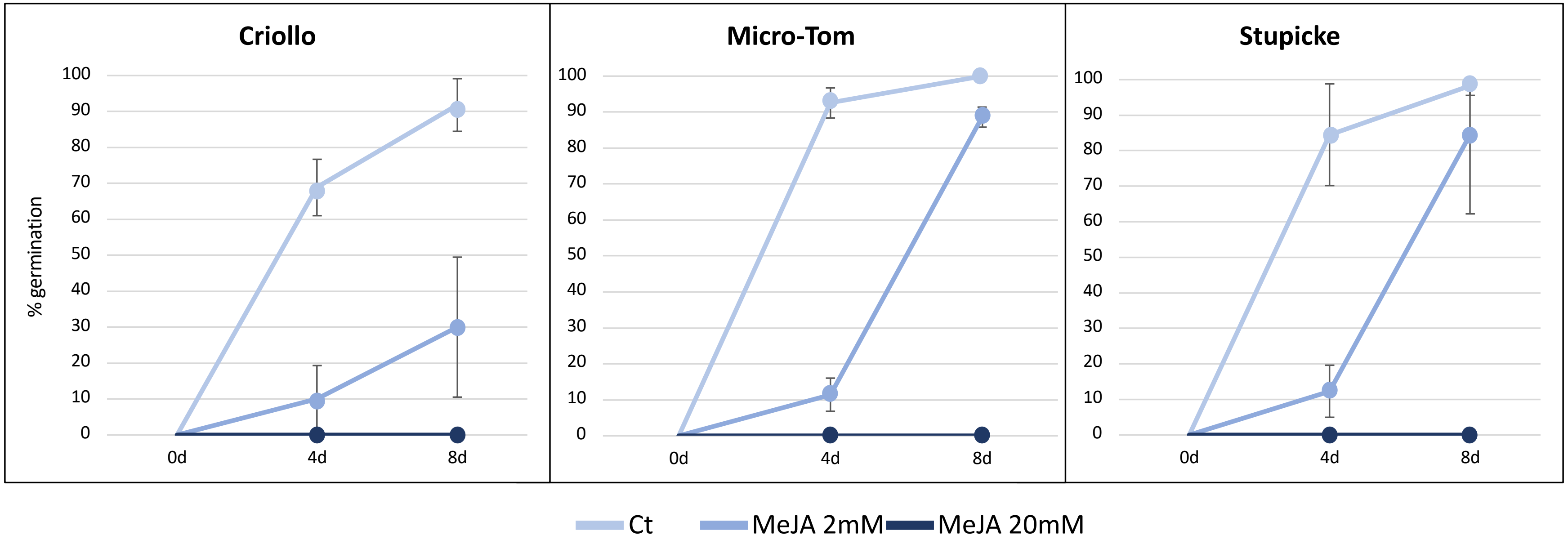

Figure S1. **Germination assay on tomato seeds primed with MeJA.** Three biological replicates of 30 seeds were used to assess the effect of MeJA osmopriming on germination rate of the three genotypes Micro-Tom, Criollo and Stupicke. Lines of differen colors indicate the different MeJA dosages used for the osmopriming: Ct, mock controlo without MeJA; MeJA 2, MeJA 2 mM; MeJA 20, MeJA 20 mM. Seeds were placed in sterile glass Petri dishes with sterile Whatman filter paper imbimbed in milliQ sterile water. Germination percentage was assessed at 4 and 8 days after sowing. The experiment was repeated two times with consistent results. Error bars represent SD.
