# Supplementary material for: Antimicrobial Peptides as Part of the Arsenal of Constitutive and Inducible Seed Defences in Tomato Seed Exudates Against Pathogens
Source: Mol Plant Pathol. 2025 Oct 27;26(10):e70164. doi: 10.1111/mpp.70164 (PMC12558808; doi:10.1111/mpp.70164)
Supplement: Supplementary file 2 — Figure S2: Distribution of differentially accumulated metabolites (DAMs) induced by methyl jasmonate (MeJA) treatment from all genotypes. Left, pie chart showing the distribution of DAMs increasingly accumulated following MeJA treatment from all three genotypes combined across metabolic pathways. Right, table resuming their number and providing the colour key for the pie chart. [file MPP-26-e70164-s002.pdf]

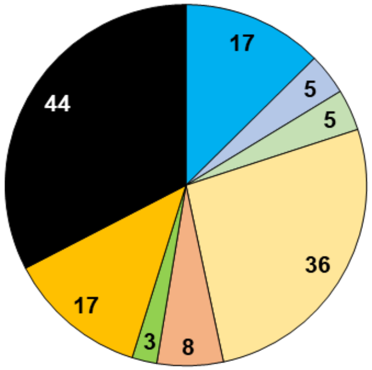

Biochemical category	
Alkaloids	17
Amino acids and peptides	5
Carbohydrates	5
Fatty acids	36
Fatty acyl glycosides	8
Shikimates and Phenylpropanoids	3
Terpenoids	17
Not Annotated	44
**TOTAL**	**135**

Figure S2. **Distribution of Differentially Accumulated Metabolites induced by MeJA treatment from all genotypes.**
Left, pie chart showing the distribution of DAMs increasingly accumulated following MeJA treatment from all three genotypes combined across metabolic pathways. Right, table resuming their number and providing the colour key for the pie chart.
