# Supplementary material for: Antimicrobial Peptides as Part of the Arsenal of Constitutive and Inducible Seed Defences in Tomato Seed Exudates Against Pathogens
Source: Mol Plant Pathol. 2025 Oct 27;26(10):e70164. doi: 10.1111/mpp.70164 (PMC12558808; doi:10.1111/mpp.70164)
Supplement: Supplementary file 3 — Figure S3: Response to damping‐off of primed tomato seeds. Seeds of Micro‐Tom, Criollo and Stupicke genotypes primed with polyethylene glycol (PEG) 8000 plus β‐aminobutyric acid (BABA) 10 mM (BABA), MeJA 2 mM (methyl jasmonate) or mock (Ct) were sown in trays containing soil inoculated with Pythium ultimum (yellow bars, condition ‘Pythium’) or mock‐inoculated (green bars, condition ‘without Pythium’). Germination rates after 14 days were computed as emergency probabilities. The experiment was repeated three times with consistent results, with n = 20. Error bars represent 95% confidence intervals. [file MPP-26-e70164-s008.pdf]

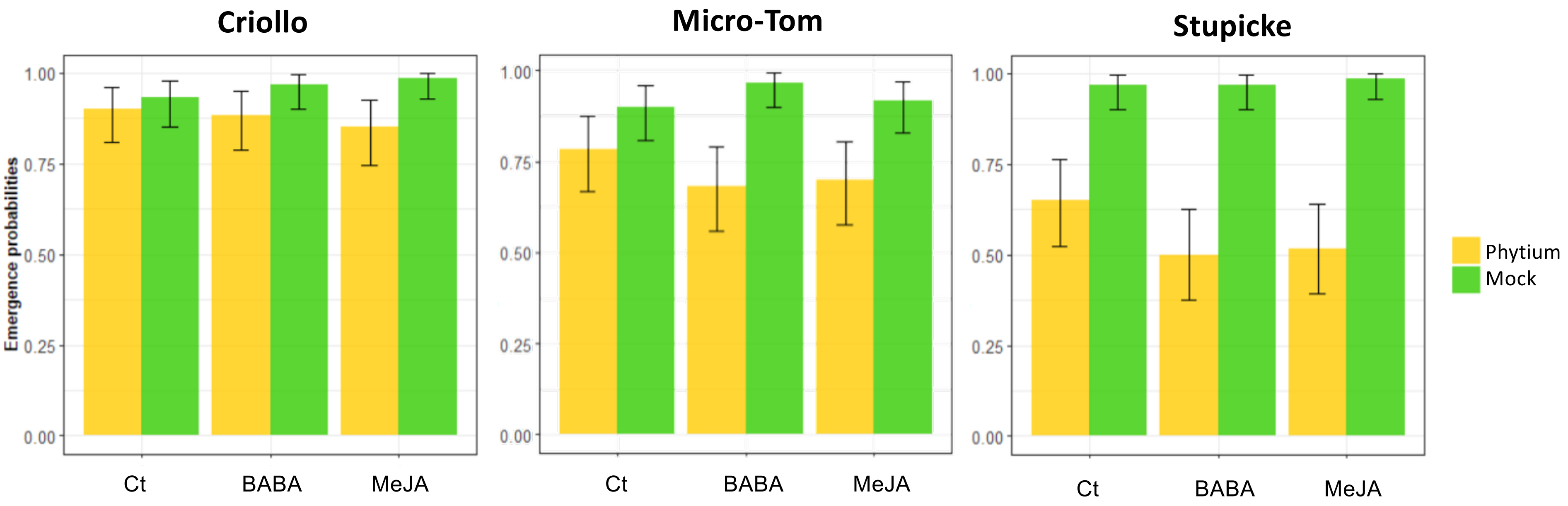

Figure S3. **Response to damping-off of primed tomato seeds.** Seeds of Micro-Tom, Criollo and Stupicke genotypes primed with PEG 8000 plus BABA 10 mM (BABA), MeJA 2 mM (MeJA) or mock (Ct) were sown in trays containing soil inoculated with *P. ultimum* (yellow bars, condition 'Pythium') or mock-inoculated (green bars, condition 'Mock'). Germination rates after 14 d were computed as emergency probabilities. The experiment was repeated three times with consistent results, with n=20. Error bars represent 95% confidence intervals.
